# Requirements for nuclear GRP78 transcriptional regulatory activities and interaction with nuclear GRP94

**DOI:** 10.1016/j.jbc.2025.108369

**Published:** 2025-02-28

**Authors:** Ze Liu, Dat P. Ha, Liangguang Leo Lin, Ling Qi, Amy S. Lee

**Affiliations:** 1 Department of Biochemistry and Molecular Medicine, Keck School of Medicine, University of Southern California, Los Angeles, California, USA; 2 Norris Comprehensive Cancer Center, Keck School of Medicine, University of Southern California, Los Angeles, California, USA; 3 Department of Molecular Physiology and Biological Physics, University of Virginia, School of Medicine, Charlottesville, Virginia, USA

**Keywords:** GRP78, GRP94, ER stress, ERAD, nuclear translocation, transcriptional regulation, ID2

## Abstract

GRP78, a molecular chaperone primarily located in the endoplasmic reticulum (ER), has recently been discovered to translocate into the nucleus of stressed and cancer cells where it assumes a new function reprogramming the transcriptome. This study explores the requirements of GRP78 nuclear translocation and its transcriptional activity and investigates the role of ER-associated degradation in the process. We show that the ER-processed, mature form of GRP78 is the major form of nuclear GRP78 and is the form with transcriptional regulatory activity. In contrast, exogenously expressed GRP78 designed to lack its ER signal peptide, thus preventing it from entering the ER or undergoing any ER-related processing/modification, while able to enter the nucleus, lacks transcriptional regulatory activity toward E-Box containing target genes. Additionally, the ATP-binding and substrate-binding activities of GRP78 are critical for this transcriptional regulatory function. We further discover that GRP94, an ER chaperone that acts in concert with GRP78 on protein folding, can translocate to the nucleus and colocalize with nuclear GRP78 upon ER stress. These findings suggest that some form of ER processing of GRP78, in addition to cleavage of the ER signal peptide, is critical for its nuclear activity and that in stressed cells, ER chaperones may assume new functions in the nucleus yet to be explored.

Molecular chaperones are essential cellular proteins that facilitate protein folding, assembly, and degradation, thereby maintaining proteostasis (1, 2). Among them, the 78-kDa glucose-regulated protein (GRP78), also referred to as BiP, is a member of the heat shock protein 70 family and primarily resides in the endoplasmic reticulum (ER), where it plays pivotal roles in protein folding and quality control (3, 4). In addition, GRP78 is a key regulator of the unfolded protein response (UPR), an evolutionarily conserved adaptive mechanism that helps cells mitigate proteotoxic stress, which is often associated with oncogenic transformation, metabolic imbalances, and neurodegenerative diseases (5, 6, 7, 8, 9).

While nonmembrane bound ER chaperones such as GRP78 is traditionally regarded as ER luminal proteins, emerging evidence indicates that ER stress not only elevates their expression but also promotes their translocation to other cellular compartments, where they acquire novel regulatory functions (10, 11, 12). For example, upon ER stress, GRP78 is actively translocated to the cell surface *via* disruption of the Golgi-ER retrograde transport or endosomal trafficking in specific cell types, and functions as a coreceptor impacting various cellular signaling pathways, as well as viral internalization (13, 14, 15, 16). Recently, we discovered that GRP78 contains a nuclear localization signal (NLS) and can translocate to the nucleus in both stressed and malignant cells, where it binds the transcriptional repressor ID2, a basic helix-loop-helix (bHLH) transcriptional factor which functions as a dominant negative inhibitor of E proteins with tumor suppressor properties in lung cancer (17). Upon binding to GRP78, ID2 is sequestered away from inhibiting E protein and a basic helix-loop-helix interaction, leading to transcription activation of the downstream target genes toward a more invasive and migratory phenotype. While the discovery that upon stress, GRP78 can translocate to the nucleus and serve as a transcriptional regulator capable of reprogramming the cells' transcriptome opens up new frontiers in our understanding of chaperone regulation and function, it also raises several critical questions.

First, where is the origin of the nuclear form of GRP78? GRP78 contains an ER signal peptide, which directs it to the ER and upon cleavage, GRP78 is delivered into the ER lumen. ER stress could overwhelm the ER processing system leaving behind cytosolic unprocessed GRP78 containing an intact NLS that allows its nuclear entry. Alternatively, the nuclear form could derive from the ER processed form. However, since there is no direct nuclear translocation machinery that connects the ER lumen to the nucleus, in that scenario GRP78 must first exit from the ER to the cytosol prior to nuclear entry. If so, how does GRP78 reflux from the ER to the cytosol?

ER-associated degradation (ERAD) is the major efflux mechanism for transporting proteins from the ER to the cytosol. This highly conserved and constitutively active process is best known to target misfolded proteins in the ER for the cytosolic proteasomal degradation (18, 19). Among various ERAD complexes, the SEL1L-HRD1 protein complex is the most evolutionarily conserved (18, 19). Recent studies using cell type-specific SEL1L or HRD1 KO mouse models have highlighted the pathophysiological significance of the SEL1L-HRD1 ERAD pathway in a substrate-specific manner (18, 20, 21). Notably, a deficiency in ERAD provides protection against virus-induced cell death (22). While the exact mechanism is not fully understood, one possibility is that SEL1L-HRD1 ERAD regulates viral entry into the cytosol. Furthermore, a recent study revealed that properly folded ER proteins can reflux into the cytosol during ER stress, and this process is exacerbated by ERAD deficiency (23).

Second, as an ER chaperone, GRP78 possesses several functional domains including the nucleotide binding domain at the N terminus which binds nucleotides like ATP and acts as an ATPase domain and the substrate binding domain which interacts directly with unfolded proteins (3). Additionally, there are co-chaperone interaction sites located on the nucleotide binding domain and substrate binding domain where they can modulate the activity of these domains (24). Which of these domains and interaction sites are required for its nuclear entry, its binding to ID2, and its transcriptional regulatory activity? Third, other ER chaperones such as GRP94 are known to form complex with GRP78 to perform their ER functions (25). Since GRP94 also contains a NLS (17), can it translocate to the nucleus and form complex with GRP78? In this study, we sought to address these critical questions utilizing a combination of biochemical, mutational, and imaging approaches. Our studies uncovered a novel interaction between GRP78 and GRP94 in the nucleus upon ER stress and revealed that ER processing of GRP78, along with its ATP-binding and substrate-binding activities, are required for its nuclear transcriptional regulatory function and complex formation with GRP94. Importantly, these new findings reveal that SEL1L-HRD1 ERAD deficiency, which upregulates GRP78 levels, enhances nuclear translocation of GRP78 and further suggest upon ER stress, ER chaperones, which are well studied inside the ER compartment, may also translocate to the nucleus assuming new functions yet to be explored.

## Results

### Nuclear GRP78 majorly consists of the ER-processed, mature form of GRP78

**Figure 1:**
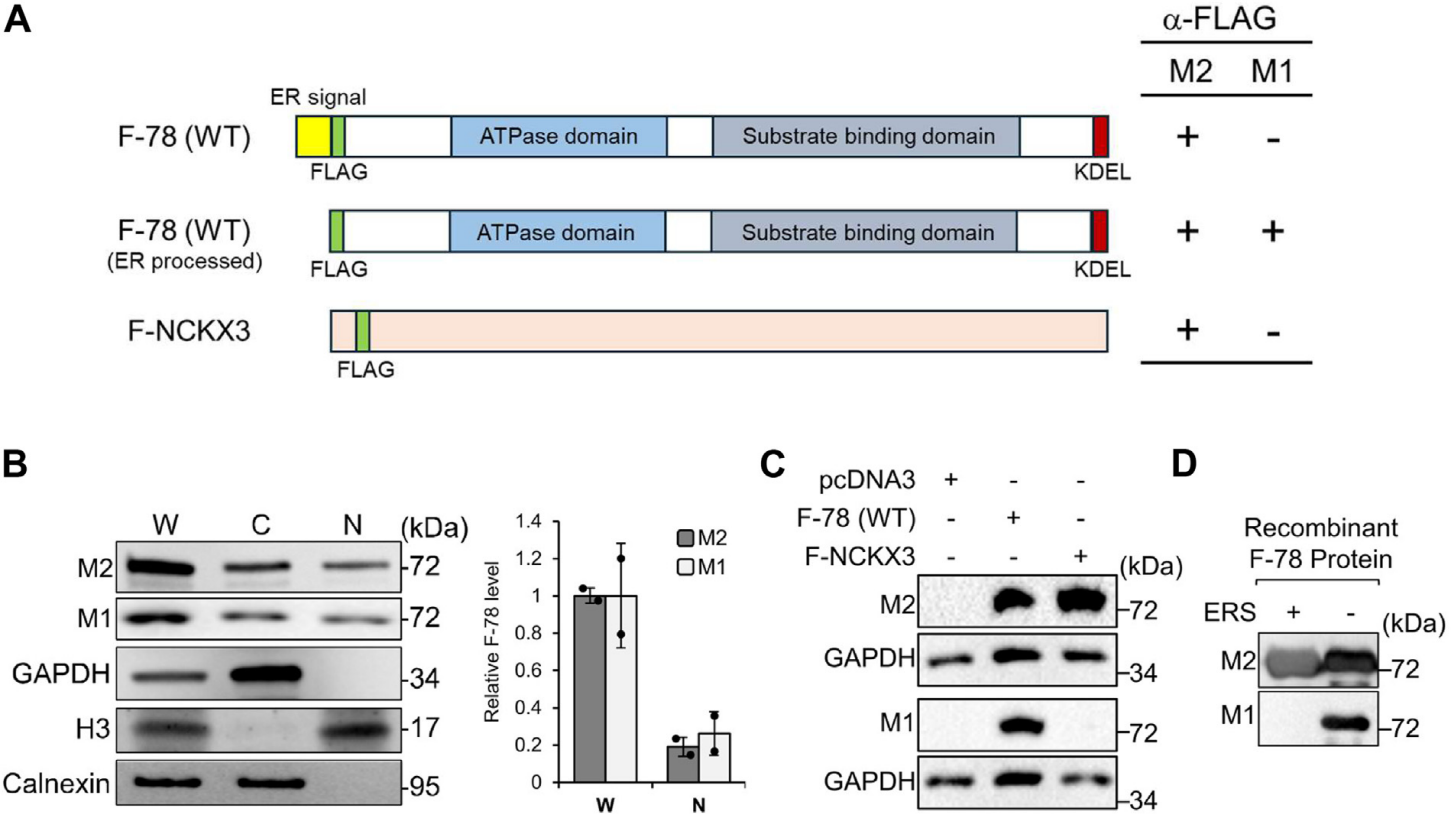
**Nuclear GRP78 majorly consists of the processed form of GRP78.***A*, schematic drawings of the FLAG-GRP78 (F-78) construct, with the unprocessed (*top*) or ER processed form (*middle*) and the FLAG-NCKX3 construct (*bottom*) showing the position of the FLAG tag. The ability of the anti-FLAG antibodies (M2 and M1) to recognize each form of GRP78 and NCKX3 is outlined on the *right*. *B*, Western blot of whole cell lysate (W), cytoplasmic (C), and nuclear (N) fractions of HEK293AD cells transfected with the expression vector for F-78 (WT) for 48 h. The fractions were separated on 10% SDS-PAGE gel and the Western blot was performed using two anti-FLAG antibodies (M2 and M1), with GAPDH, histone H3, and calnexin serving as cytoplasmic, nuclear, and ER markers respectively (n = 2). The FLAG protein bands detected by the M2 and M1 antibodies in W and N fractions were quantitated, normalized against H3, and the relative levels in W and N fractions are plotted. Data are presented as mean ± S.D. *C*, Western blot of HEK293AD cells transfected with empty vector pcDNA3, the expression vector for F-78 (WT) or F-NCKX3 for 48 h and probed with the M2 or M1 antibody with GAPDH serving as loading control. *D*, Western blot of recombinant F-78 proteins, with or without the ER signal peptide (ERS) expressed in *Escherichia coli* and probed with the M2 or M1 antibody (n = 2). ER, endoplasmic reticulum.

GRP78, as an ER-resident chaperone, is processed in the ER, where its signal peptide is cleaved to yield the mature protein. To test if ER processing is necessary for GRP78 nuclear translocation, we transfected human embryonic kidney HEK293AD cells with an expression vector for FLAG-tagged wild type GRP78 [F-78 (WT)], where the FLAG epitope was inserted immediately after the ER signal peptide (Fig. 1*A*). The cell lysate was subjected to subcellular fractionation to isolate the cytoplasmic and nuclear fractions, followed by Western blot analysis. In the nuclear fraction, there was no detectable contamination from the ER as indicated by the absence of the ER transmembrane protein calnexin (Fig. 1*B*). As expected, histone H3 protein was present in the nuclear fraction but absent in the cytoplasmic fraction (Fig. 1*B*). For detection of F-78, we utilized two monoclonal anti-FLAG antibodies (M2 and M1), with different binding properties for the FLAG epitope. The M2 antibody recognizes the FLAG peptide sequence at the N terminus, Met-N terminus, C terminus, and internal sites of the fusion protein, whereas the M1 antibody binds to the FLAG-epitope when it is located at the free N terminus of a fusion protein, but not Met-FLAG fusion proteins, so will not recognize unprocessed proteins expressed in the cytoplasm. Thus, as summarized in Figure 1*A*, the M2 antibody is expected to bind both F-78 (WT) as well as its ER processed form, whereas the M1 antibody will only recognize its ER-processed form with the FLAG-epitope at the free N-terminus. Our results showed that the nuclear form of GRP78 was recognized by both the M2 and M1 antibodies, indicating that the processed form of GRP78 exists in the nucleus (Fig. 1*B*). Furthermore, both the M2 and M1 antibodies detected similar levels of GRP78 in nucleus, suggesting that nuclear GRP78 majorly consists of the ER-processed, mature form of GRP78.

To independently validate the specificity of the M1 antibody for recognizing FLAG-epitope exclusively at the free N terminus, but not at an internal site, of a fusion protein, we employed two complementary approaches. First, we utilized an expression vector for FLAG-NCKX3, a protein with a known internally inserted FLAG-epitope (26), to serve as an independent control unrelated to F-78 (Fig. 1*A*). HEK293AD cells were transfected with the pcDNA3 empty vector, the F-78 (WT) expression vector with the FLAG tag at the free N terminus after ER processing, or the F-NCKX3 expression vector with an internal FLAG tag. Western blot analysis revealed that the M2 antibody recognized both F-78 (WT) and F-NCKX3, consistent with its known ability to detect internal and terminal FLAG epitopes (Fig. 1*C*). In contrast, the M1 antibody selectively recognized F-78 (WT) but not F-NCKX3, confirming its specificity for FLAG-epitope at the free N terminus but not internal FLAG (Fig. 1*C*).

Second, to directly test that the M1 antibody only binds GRP78 with the FLAG-tag exposed at the N terminus but not the unprocessed form still containing the ER signal peptide preceding the FLAG-tag, we designed and obtained two recombinant FLAG-tagged GRP78 proteins produced in *Escherichia coli* (Fig. S1). The first recombinant protein F-78 (+ERS) included the ER signal peptide of GRP78 (amino acids 1–18) with a FLAG-tag inserted immediately after the ER signal peptide (Fig. S1*A*). In mammalian cells, the ER signal peptide is cleaved during translation into the ER, resulting in mature GRP78 with an N-terminal FLAG-epitope. However, in *E. coli*, which lacks the ER, the signal peptide remains uncleaved, leaving the FLAG-tag internal. The second recombinant protein F-78 (ΔERS) was designed with the ER signal peptide removed, positioning the FLAG tag at the N terminus to mimic the cleaved form of GRP78 (Fig. S1*B*). Western blot analysis of these recombinant proteins showed that the M2 antibody recognized both F-78 (+ERS) and F-78 (ΔERS), consistent with its ability to detect FLAG-epitopes regardless of location in the fusion protein (Fig. 1*D*). In contrast, the M1 antibody only recognized F-78 (ΔERS) but not F-78 (+ERS), validating its specific binding to F-78 devoid of its ER signal peptide (Fig. 1*D*). Collectively, the results confirm the specificity of the anti-FLAG M2 and M1 antibodies utilized in this analysis.

### GRP78 nuclear translocation is enhanced by SEL1L-HRD1 ERAD deficiency

**Figure 2:**
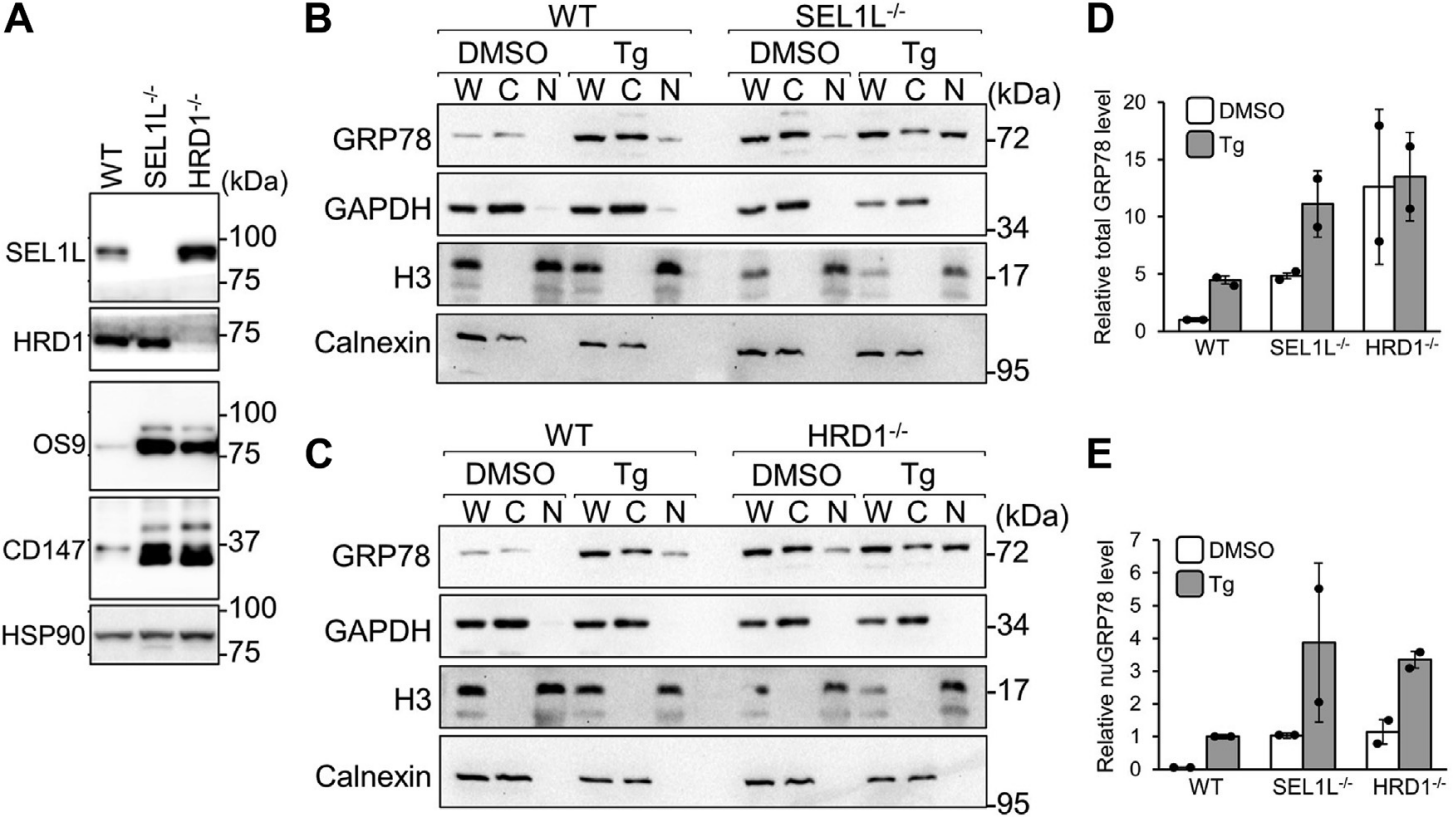
**GRP78 nuclear translocation is enhanced by ERAD deficiency.***A*, WT, SEL1L^−/−^, and HRD1^−/−^ HEK293T cell lysates were analyzed by Western blot for the indicated protein with HSP90 serving as loading control. *B*, WT and SEL1L^−/−^ HEK293T cells were treated with DMSO or Tg for 24 h followed by subcellular fractionation. The whole cell lysate (W), cytoplasmic (C), and nuclear (N) fractions were analyzed by Western blot for GRP78 protein level with GAPDH, H3, and calnexin serving as cytoplasmic, nuclear, and ER markers respectively (n = 2). *C*, same as in (*B*) except WT and HRD1^−/−^ HEK293T cells were used (n = 2). *D*, the total GRP78 protein levels (detected in the W fraction) in (*B* and *C*) for the three cell lines, either DMSO or Tg-treated were quantitated, normalized against GAPDH, and their relative levels are plotted. *E*, the nuclear GRP78 (nuGRP78) protein levels (detected in the N fraction) in (*B* and *C*) for the three cell lines, either DMSO or Tg-treated were quantitated, normalized against H3, and their relative levels are plotted. Data are presented as mean ± S.D. DMSO, dimethyl sulfoxide; ER, endoplasmic reticulum; ERAD, ER-associated degradation; Tg, thapsigargin.

A potential mechanism for GRP78 to efflux from the ER to the cytosol is through ERAD. The SEL1L-HRD1 complex represents one of the most conserved ERAD pathways (27, 28, 29). We generated SEL1L or HRD1 ERAD-deficient HEK293T cells using the CRISPR/Cas9 system and validated by the accumulation of two known endogenous ERAD substrates, OS9 and CD147 (30) (Fig. 2*A*). The SEL1L or HRD1 KO cells were then treated with the ER stress inducer thapsigargin (Tg) followed by subcellular fractionation and Western blot analysis. Our results showed that in WT cells, in the absence of ER stress, no GRP78 was detected in the nuclear fraction, and upon Tg treatment, total GRP78 level was upregulated by about 5-fold and nuclear GRP78 was detected (Fig. 2, *B*–*E*). For the SEL1L and HRD1 KO cells, it was previously reported that KO of either gene by itself upregulated GRP78 (31, 32). In agreement, we observed that in dimethyl sulfoxide (DMSO)-treated SEL1L and HRD1 KO cells, total GRP78 protein levels were elevated by 5- and 12-fold respectively compared to DMSO-treated WT cells (Fig. 2, *B*–*D*). Upon Tg treatment, total GRP78 level was increased by about 2- fold in the SEL1L KO cells and only increased minimally in the HRD1 KO cells (Fig. 2, *B*–*D*). For WT cells, nuclear GRP78 was detected upon Tg treatment but not in the DMSO-treated cells. In contrast, nuclear GRP78 was detected in DMSO-treated SEL1L and HRD1 KO cells (Fig. 2, *B*, *C*, and *E*). Strikingly, compared to WT cells, there was a 3.9- and 3.3-fold increase in nuclear GRP78 level in SEL1L and HRD1 KO cells, respectively, in the Tg-treated cells (Fig. 2*E*).

### ER processing of GRP78 is necessary for its nuclear transcriptional regulatory activity

**Figure 3:**
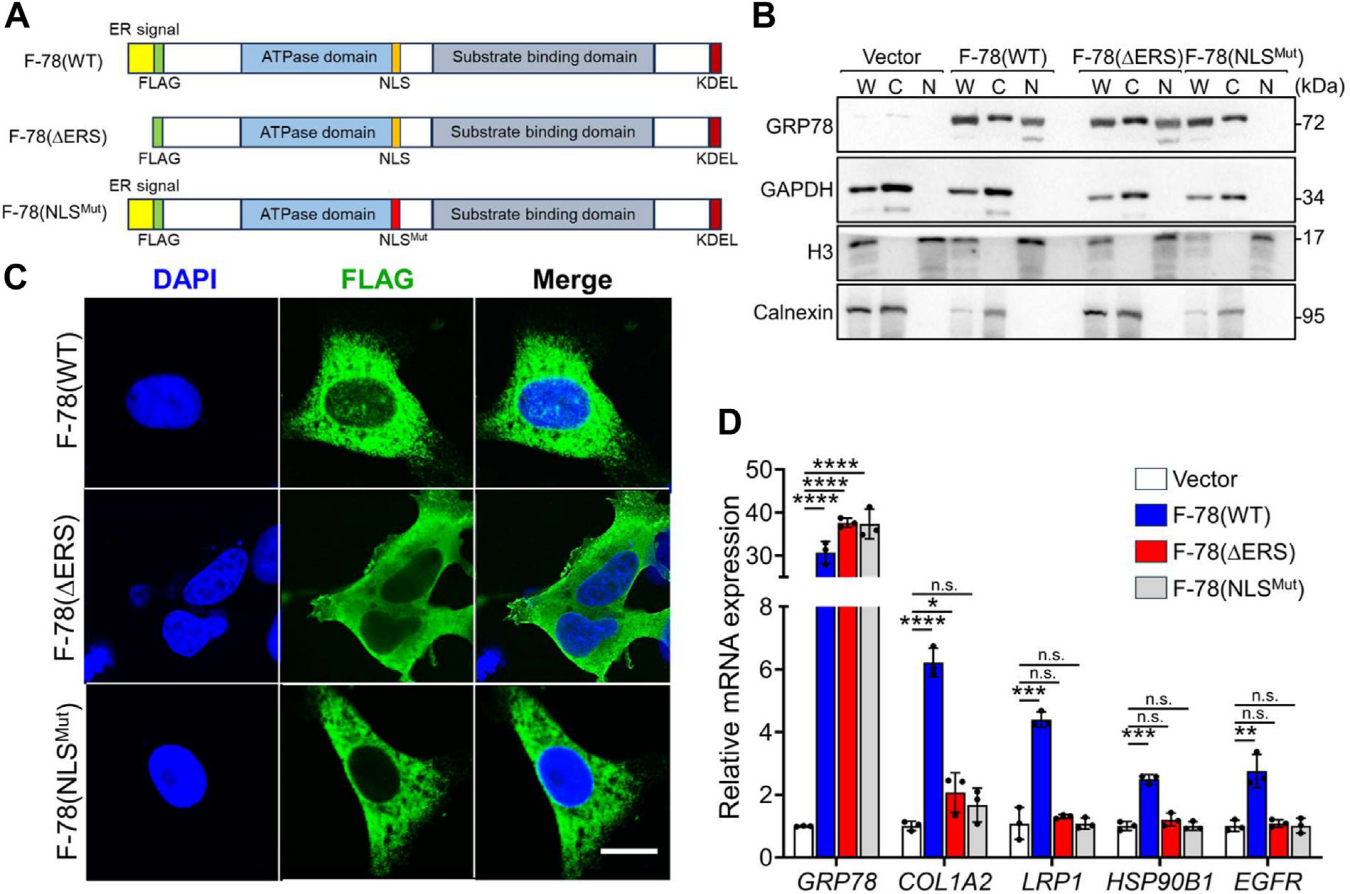
**Requirement of the ER signal peptide of GRP78 for its nuclear transcriptional regulatory activity.***A*, schematic drawing of the F-78 (WT), ΔERS, and NLS^Mut^ constructs. *B*, HEK293AD cells were transfected with empty vector, expression vector for F-78 (WT), or F-78 mutants (ΔERS, or NLS^Mut^) for 48 h followed by subcellular fractionations. The whole cell lysate (W), cytoplasmic (C), and nuclear (N) fractions were analyzed by Western blot for GRP78 protein level with GAPDH, H3, and calnexin serving as cytoplasmic, nuclear, and ER markers, respectively. *C*, representative confocal immunofluorescence images of HEK293AD cells transfected with expression vectors for F-78 (WT) (*top*), F-78 (ΔERS) (*middle*), or F-78 (NLS^Mut^) (*bottom*) for 48 h. The nuclei were stained by DAPI in *blue*. The scale bars represent 10 μm. *D*, RT-qPCR analysis of *GRP78*, *COL1A2*, *LRP1*, *HSP90B1*, and *EGFR* mRNA levels normalized to *β-actin* in HEK293AD cells transfected with empty vector, expression vectors for F-78 (WT), F-78 (ΔERS), or F-78 (NLS^Mut^) for 48 h (n = 3). Data are presented as mean ± S.D. ∗*p* ≤ 0.05, ∗∗*p* ≤ 0.01, ∗∗∗*p* ≤ 0.001, ∗∗∗∗*p* ≤ 0.0001, n.s. denotes not significant (Student's *t* test, Bonferroni correction). DAPI, 4′,6-diamidino-2-phenylindole; ER, endoplasmic reticulum; NLS, nuclear localization signal.

Previously, we have demonstrated that nuclear GRP78 can transcriptionally activate a subset of genes containing E-Box element in their promoter. The ER signal peptide of GRP78 directs its insertion into the ER to be processed to generate the mature protein. To test the requirement of this process in the transcriptional regulatory activity of nuclear GRP78, we constructed a FLAG-GRP78 mutant designed to lack the ER signal peptide [F-78 (ΔERS)], thus preventing it from entering the ER and remains cytosolic upon synthesis. While it retains an intact nuclear localization signal allowing it to translocate into the nucleus, in contrast to the ER-processed, mature form of GRP78, F-78 (ΔERS) has not been subjected to ER-related processing or modification prior to nuclear entry. F-78 (WT) was used as a positive control for nuclear entry. For negative control, we utilized a FLAG-GRP78 mutant where the NLS adjacent to the ATPase domain has been mutated [F78 (NLS^Mut^)] (Fig. 3*A*). As previously described, in this mutant, the three lysine residues (K276, K280, and K287) within the NLS were mutated to alanine, thereby lowering its NLS score and preventing it from translocating into the nucleus (17). We transfected these constructs into HEK293AD cells followed by fractionation and Western blot analysis. The results showed that F-78 (ΔERS) containing the NLS can translocate to the nucleus similar to F-78 (WT), whereas the F78 (NLS^Mut^) was not detected in the nucleus as expected (Fig. 3*B*). Furthermore, immunofluorescence confocal microscopy revealed that F-78 (WT) formed discrete nuclear foci, while F-78 (ΔERS) exhibited diffused nuclear staining pattern, and the F78 (NLS^Mut^) was absent from the nucleus (Fig. 3*C*).

We then investigated the transcriptional regulatory activities of these three constructs by transfecting them into HEK293AD cells followed by RNA extraction and reverse transcription-quantitative PCR (RT-qPCR) analysis to measure transcript levels of known nuclear GRP78-regulated targets (17). While F-78 (WT) upregulated the known target genes, both the ΔERS and NLS mutants failed to do so (Fig. 3*D*). These findings suggest that while the NLS is critical for nuclear translocation, ER processing of GRP78 is necessary for its nuclear transcriptional regulatory activity and that nuclear foci correlate with transcriptional regulation.

### Mutational analysis of GRP78 for its nuclear localization, ID2 interaction, and transcriptional regulation

**Figure 4:**
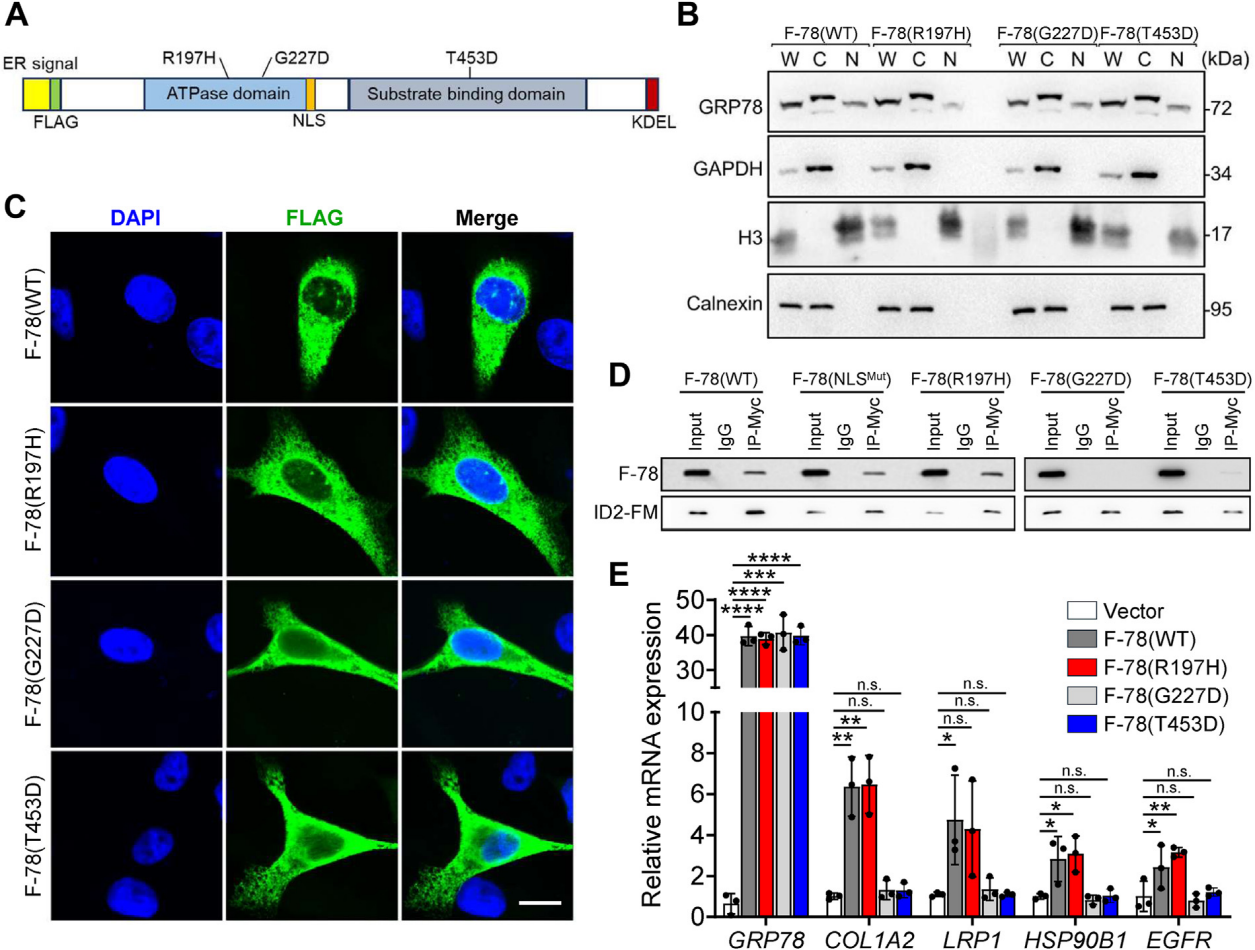
**Mutational analysis of GRP78 for its nuclear translocation and its transcriptional regulatory activity.***A*, schematic drawing of the F-78 with the functional domains and positions of three different point mutations (R197H, G227D, and T453D) indicated. *B*, HEK293AD cells were transfected with expression vectors for F-78 (WT) or the indicated F-78 mutants (R197H, G227D, or T453D) for 48 h followed by subcellular fractionations. The whole cell lysate (W), cytoplasmic (C), and nuclear (N) fractions were analyzed by Western blot for GRP78 protein level with GAPDH, H3, and calnexin serving as cytoplasmic, nuclear, and ER markers respectively. *C*, representative confocal immunofluorescence images of HEK293AD cells transfected with expression vectors for F-78 WT or the indicated F-78 mutants (R197H, G227D, or T453D) for 48 h. The nuclei were stained by DAPI in *blue*. The scale bars represent 10 μm. *D*, HEK293AD cells were cotransfected with the expression vectors for ID2-FLAG-Myc (ID2-FM) and either F-78 (WT) or F-78 mutants (NLS^Mut^, R197H, G227D, or T453D) and coimmunoprecipitation assays were performed using IgG or anti-Myc antibodies. The immunoprecipitated proteins were probed for F-78 and ID2-FM using the anti-FLAG (M2) antibody. *E*, RT-qPCR analysis of *GRP78*, *COL1A2*, *LRP1*, *HSP90B1*, and *EGFR* mRNA levels normalized to *β-actin* in HEK293AD cells transfected with empty vector or expression vectors for F-78 (WT) and F-78 mutants (R197H, G227D, or T453D) for 48 h (n = 3). Data are presented as mean ± S.D. ∗*p* ≤ 0.05, ∗∗*p* ≤ 0.01, ∗∗∗*p* ≤ 0.001, ∗∗∗∗*p* ≤ 0.0001, n.s. denotes not significant (Student's *t* test, Bonferroni correction). DAPI, 4′,6-diamidino-2-phenylindole; ER, endoplasmic reticulum; IgG, immunoglobulin G.

To investigate GRP78 interaction sites required for its nuclear translocation and function, we utilized three FLAG-GRP78 mutants: R197H (cochaperone binding deficient), G227D (ATP binding deficient), and T453D (substrate binding deficient) (Fig. 4*A*). As reported previously, the R197H mutant only impairs interaction between GRP78 and ER-localized DnaJ-domain-containing proteins and does not show global functional defects, as indicated by its WT basal ATPase rate, its ability to undergo peptide-stimulated ATPase hydrolysis and its WT proteolytic pattern in the presence of ADP or ATP (24, 33). The G227D mutant exhibits reduced binding affinity for ATP while maintaining its ability to bind peptides (34). The T453D mutant is a client-binding GRP78 mutant, similar to the yeast Kar2p substrate-binding mutant *Kar2-133* (35). These mutants along with F-78 (WT) were transfected into HEK293AD cells followed by subcellular fractionation and Western blot analysis. We observed that all three mutants, which still contain the NLS, were able to translocate to the nucleus (Fig. 4*B*). Immunofluorescent staining confirmed nuclear localization for all mutants, though only the WT and R197H mutant displayed discrete nuclear foci, while G227D and T453D mutants exhibited diffuse patterns similar to F-78 (ΔERS) (Fig. 4*C*).

Next, we determined if these GRP78 mutants can interact with ID2, a nuclear transcriptional repressor, which has been previously shown to bind nuclear GRP78 and mediates its transcriptional regulatory function (17). In co-immunoprecipitation experiments, we observed that ID2-FLAG-Myc (ID2-FM) was able to immunoprecipitate F-78 (WT), R197H, and NLS^Mut^ mutants but not the G227D and T453D mutants (Fig. 4*D*). RT-qPCR analysis further demonstrated that while the F-78 (WT) and mutant vectors were expressed in similar amount, only F-78 (WT) and R197H, but not G227D and T453D, were able to upregulate downstream genes controlled by nuclear GRP78 (Fig. 4*E*). These findings indicate that while all the mutant GRP78 proteins being tested can still enter the nucleus, GRP78 transcriptional activity requires its ATP-binding and substrate-binding activities.

### ER chaperone GRP94 translocates to the nucleus under ER stress and forms complex with nuclear GRP78

**Figure 5:**
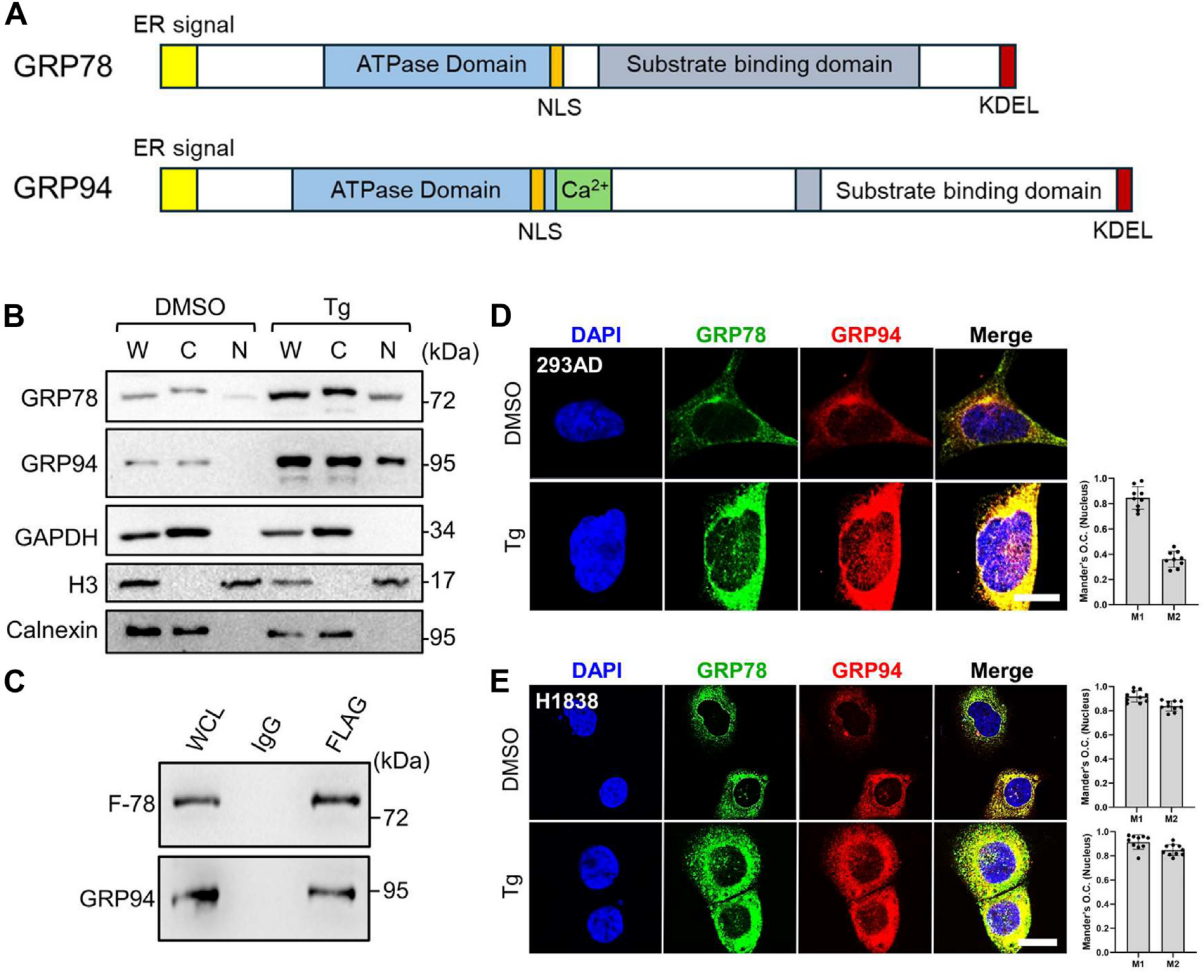
**ER chaperone GRP94 translocates to the nucleus under ER stress and forms complex with nuclear GRP78.***A*, schematic drawing of endogenous GRP78 (*top*) and GRP94 (*bottom*) proteins with the functional domains indicated. *B*, HEK293AD cells were treated with DMSO or Tg for 24 h followed by subcellular fractionations. The whole cell lysate (W), cytoplasmic (C), and nuclear (N) fractions were analyzed by Western blot for GRP78 and GRP94 protein levels with GAPDH, H3, and calnexin serving as cytoplasmic, nuclear, and ER markers respectively (n = 2). *C*, HEK293AD cells were transfected with the expression vector for F-78 (WT) and coimmunoprecipitation assays were performed using IgG or anti-FLAG (M2) antibodies. The immunoprecipitated proteins were probed for F-GRP78 using the anti-FLAG (M2) antibody (*top*) or GRP94 (*bottom*) (n = 2). *D*, representative confocal immunofluorescence images of HEK293AD cells treated with DMSO or Tg for 24 h and stained for GRP78 (*green*) and GRP94 (*red*). The nuclei were stained by DAPI in *blue*. The scale bars represent 10 μm. Mander's O.C. quantification was performed for the Tg-treated cells where GRP78 and GRP94 colocalization was detected, and the values are shown on the *right*. M1 is the contribution of GRP78 to the colocalized area, whereas M2 is the contribution of GRP94. Data represent mean ± S.D. Number of analyzed independent image areas (Ar) and cells (Nu): Ar/Nu = 5/10. *E*, same as in (*D*) except human lung cancer cell H1838 was used. DAPI, 4′,6-diamidino-2-phenylindole; DMSO, dimethyl sulfoxide; ER, endoplasmic reticulum; O. C., overlap coefficient; Tg, thapsigargin.

GRP94, another major ER chaperone has previously been shown to associate with GRP78 in the ER and work in tandem to facilitate protein folding and maturation (25, 36). Interestingly, GRP94 also has an NLS in its amino acid sequence adjacent to its ATPase domain (Fig. 5*A*). To investigate whether GRP94 also translocates and colocalizes with GRP78 in the nucleus upon ER stress, HEK293AD cells were treated with Tg, followed by subcellular fractionation and Western blot analysis. We observed that both GRP78 and GRP94 were detected in the nuclear fraction of Tg-treated cells (Fig. 5*B*). Co-immunoprecipitation experiment further showed that GRP78 can form complex with GRP94 (Fig. 5*C*). Immunofluorescence staining confirmed the presence of both GRP78 and GRP94 in the nucleus in Tg-stressed HEK293AD cells (Fig. 5*D*). Strikingly, in addition to their colocalization in the ER as expected, we observed extensive colocalization between GRP78 and GRP94 in the nucleus, suggesting that they form a complex in both cellular compartments (Fig. 5*D*). The nuclear colocalization of GRP78 and GRP94 was quantified by Mander's overlap coefficient (O.C.) which showed that high percentage of GRP78 colocalized with GRP94 (high M1 value), and partial GRP94 colocalized with GRP78 (M2 value) (Fig. 5*D* right panel). Cancer cells generally upregulate GRP78 even under nonstress conditions with detectable nuclear localization of GRP78 (17). In performing immunofluorescence staining of the human lung adenocarcinoma cell line H1838, we observed that in these cancer cells, even in the absence of ER stress, both GRP78 and GRP94 could be detected in the nucleus (Fig. 5*E*). Their colocalization was substantially intensified in the Tg-treated cells both in the ER and in the nucleus (Fig. 5*E*). Quantification of their nuclear colocalization by Mander's O.C. showed that high percentage of both GRP78 and GRP94 contribute to their colocalization (Fig. 5*E* right panel).

**Figure 6:**
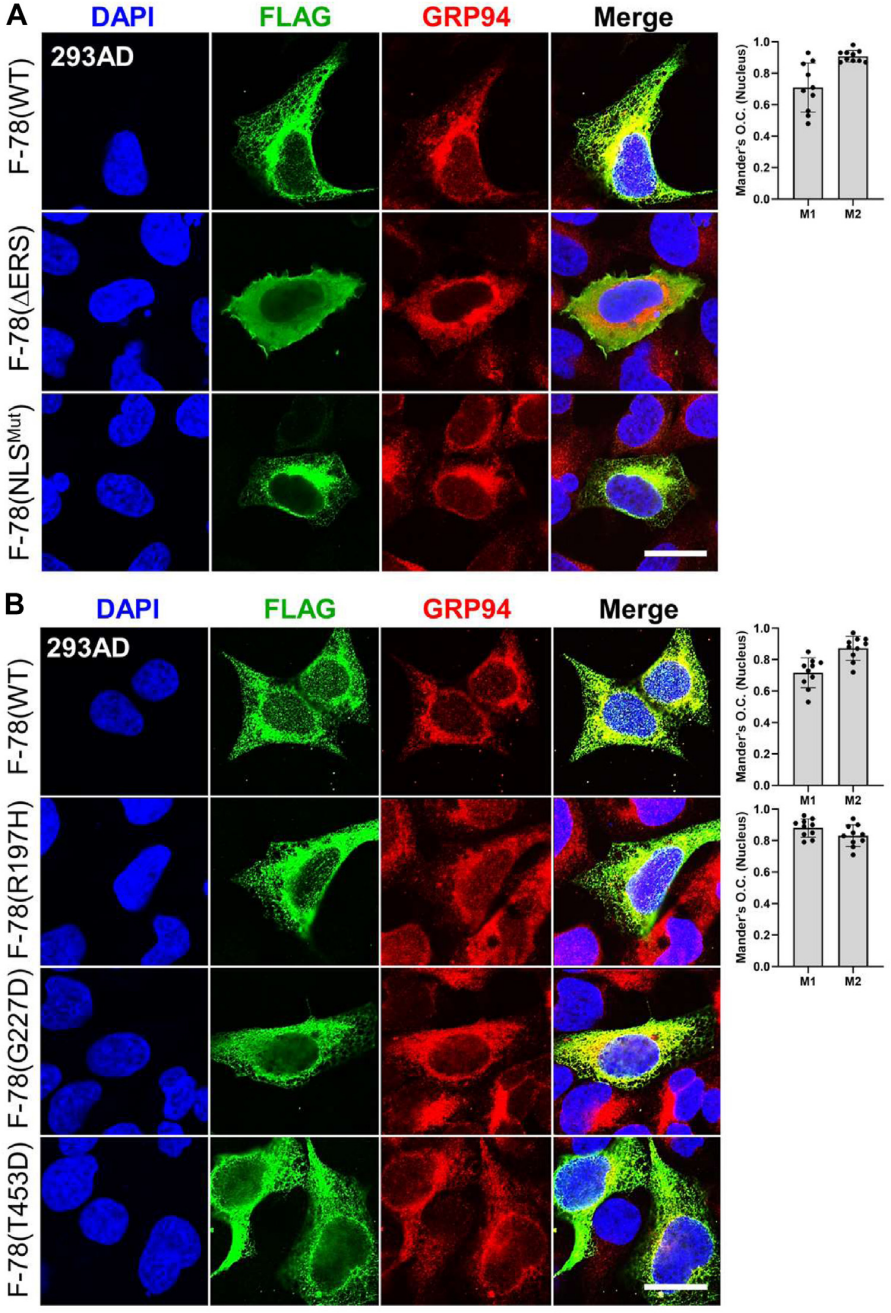
**Mutational analysis of GRP78 for its nuclear translocation and interaction with GRP94**. *A*, representative confocal immunofluorescence images of HEK293AD cells transfected with expression vectors for F-78 (WT) or the F-78 mutants (ΔERS) or F-78 (NLS^Mut^), and (*B*) transfected with expression vectors for F-78 (WT), or F-78 mutants (R197H, G227D, or T453D), for 48 h and stained for F-GRP78 using the anti-FLAG (M2) antibody (*green*) or GRP94 (*red*). The nuclei were stained by DAPI in *blue*. The scale bars represent 10 μm. For the cells showing GRP78 and GRP94 colocalization, Mander's O.C. quantification was performed, and the values are shown on the *right*. M1 is the contribution of GRP78 to the colocalized area, whereas M2 is the contribution of GRP94. Data represent mean ± S.D. Number of analyzed independent image areas (Ar) and cells (Nu): Ar/Nu = 6/10. NLS, nuclear localization signal; DAPI, 4′,6-diamidino-2-phenylindole; O. C., overlap coefficient.

**Figure 7:**
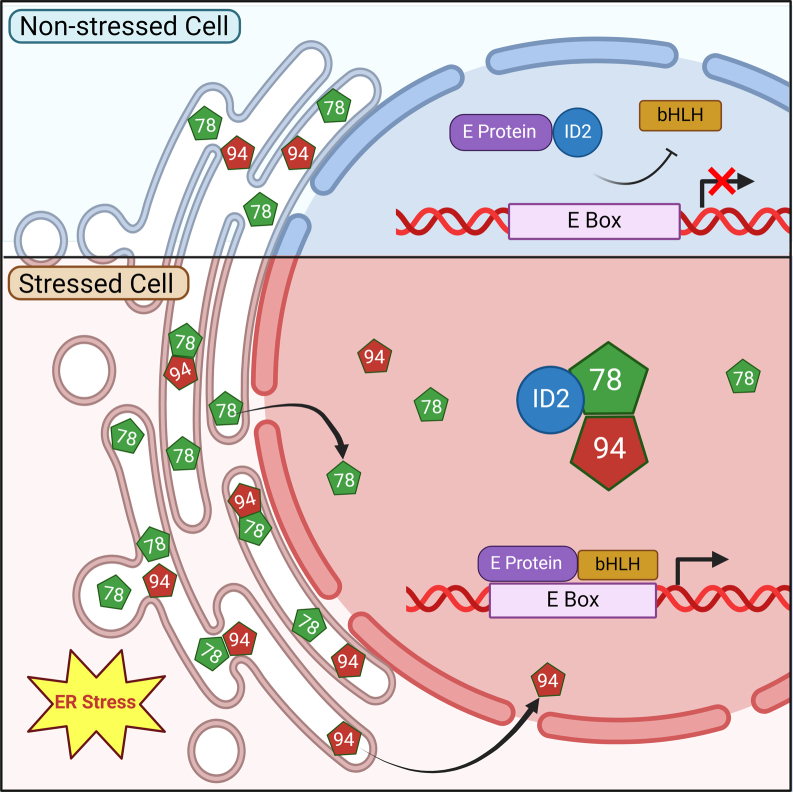
**Proposed mechanism of GRP78 nuclear translocation and its interaction with GRP94 to regulate E-Box-dependent transcriptional activity.** In nonstressed cell (*top*), ID2 binds to E protein and prevents its interaction with bHLH transcription factor. In stressed cell (*bottom*), GRP78 and GRP94 are upregulated and translocated to the nucleus where GRP78 binds and sequesters ID2, relieving the inhibitory effects on transcription leading to activation of genes. bHLH, basic helix-loop-helix.

In examining whether the GRP78 mutants described above affected the UPR or the onset of apoptosis, we noted that in nonstressed HEK293AD cells transfected with the expression vectors for WT GRP78, or mutants lacking the ER signal peptide (ΔERS), affecting its import (NLS^Mut^), or cochaperone binding activities (R197H) showed no activation of UPR markers (ATF4 and CHOP) or apoptotic markers (cleaved forms of PARP and Caspase 3). For the mutants affecting GRP78 nuclear transcriptional activity (G227D and T453D), mild increase of ATF4 and CHOP was detected, but no activation of the apoptotic markers, in contrast to ER-stressed (Tg treated) cells which showed robust activation of these markers (Fig. S2). Next, we examined whether these mutations affected their interaction with GRP94 in the nucleus. HEK293AD cells were transfected with F-78 (WT) or mutants, followed by immunofluorescence staining. Our results showed that while F-78 (WT) colocalized with GRP94 in both the ER and the nucleus, F-78 (ΔERS), which lacks the ER signal peptide, localized primarily to the cytoplasm and exhibited a diffuse nuclear staining pattern with no detectable colocalization with GRP94 in both the ER and nucleus (Fig. 6*A*). In contrast, the F-78 (NLS^Mut^) colocalized with GRP94 only in the ER, and F-78 (NLS^Mut^) was not detected in the nucleus as expected, whereas GRP94 was still detected in the nucleus (Fig. 6*A*). Furthermore, F-78 (R197H), which formed discrete nuclear foci, colocalized with GRP94 in both ER and nucleus, whereas the G227D and T453D mutants failed to form discrete foci and showed no nuclear colocalization with GRP94, although they maintained colocalization within the ER (Fig. 6*B*). For cells transfected with expression vectors for the F-78 (WT) and F-78 (R197H) which showed nuclear GRP78 and GRP94 colocalization, quantification by Mander's O.C. confirmed high percentage of both GRP78 and GRP94 contribute to their colocalization (Fig. 6, *A* and *B*, right panels). Thus, GRP94 can colocalize with GRP78 in the nucleus and that this interaction is dependent on ER processing of GRP78, as well as the ATP-binding and substrate-binding activities of GRP78. A proposed model for the translocation of GRP78 and GRP94 from the ER to the nucleus and their nuclear activities is summarized in Figure 7.

## Discussion

ER stress-mediated translocation of ER chaperones to other cellular compartments such as the cell surface, mitochondria, cytosol, and the nucleus allows cells to vastly expand their functionality beyond the ER, suggesting that the dogma of cell compartmentation might be less rigid such that intracellular plasticity is key to allow cells to adapt to stress (11, 12, 23). Our recent discovery that GRP78, a key stress-inducible ER luminal chaperone, commonly overexpressed in cancer cells and cells that have acquired therapeutic resistance, is prominently expressed in the nucleus of a wide variety of cancer cells where it acts as a transcriptional regulator allowing cells to adopt an invasive phenotype represents a paradigm shift about its role in regulating homeostasis and tumorigenesis (17). Toward understanding more fully the requirements for GRP78 nuclear translocation and its functions there, we investigated the forms of GRP78 in the nucleus, its entry process, and the contribution of GRP78 interaction sites to its nuclear activity. Our studies, which combined biochemical, mutational, and confocal microscopy, revealed several novel aspects of nuclear GRP78 that could open new frontiers in ER chaperone research.

Our results indicate that nuclear GRP78 majorly consists of the mature form of GRP78 without its ER signal peptide which has been cleaved upon ER entry, consistent with an earlier report that the form of GRP78 cross-linked to DNA in the nucleus of irradiated cells is the processed, mature form of GRP78 (37). While the precise mechanisms for the translocation of GRP78 from the ER to the nucleus remain to be determined, one scenario is that GRP78 first enters the ER, undergoes signal peptide cleavage, and subsequently exits the ER to reach the nucleus. In testing whether ERAD contributes to the flux of GRP78 from the ER to the cytosol, we observed that SEL1L and HRD1 ERAD KO cells showed upregulation of GRP78 as reported previously (31, 32), indicative of a cellular adaptation to ERAD deficiency. Notably, both ERAD KO cell lines showed a marked increase of nuclear GRP78 under ER stress. While the mechanisms involved require future investigation, it has been reported that defect in ERAD enhances chaperone efflux from the ER (23). Thus, it is possible that ERAD defect leads to an increase in misfolded proteins in the ER, which signals the efflux response. Alternatively, the SEL1L-HRD1 complex may indirectly regulate GRP78 efflux *via* a yet undefined mechanism; hence, its ablation leads to an increase in GRP78 release, rather than a decrease. Patients with SEL1L-HRD1 ERAD deficiency have recently been identified, and termed ERAD-associated neurodevelopmental disorders with onset in infancy (ENDI) (30, 38). It will be interesting to explore whether there will be higher levels of nuclear GRP78 reprogramming the transcriptome and contributing to disease progression in these patient cells. Likewise, recent reports suggest that perturbance of the proteostasis network could impact adaptive immunity as well as tumor resistance (39, 40). These could also be potential pathways affected by upregulation of nuclear GRP78 upon ERAD modulation.

In examining the various forms of GRP78 in the nucleus and their ability to activate the E-box containing target genes identified previously (17), we noted that WT GRP78 upon entry into the nucleus forms discrete foci, associating with its ability to activate the E-Box containing the target genes. In contrast, the ΔERS mutant lacking the ER signal peptide is hence synthesized and resides in the cytosol while able to enter the nucleus likely due to the intact NLS localization signal, it appears in a diffused form and fails to activate the target genes. Since the major difference between WT GRP78 and ΔERS is that the latter is unable to enter the ER and or undergo ER-related processing/modification, it is tempting to speculate that some form of ER processing of GRP78, in addition to cleavage of the ER signal peptide, is required for its transcriptional regulatory activity in the nucleus and the formation of the discrete foci. Among the GRP78 functional mutations examined, the cochaperone binding domain mutant R197H did not affect nuclear localization, discrete foci formation, or transcriptional activity on the target genes, hinting that the cochaperone binding activity of GRP78 may not be critical for its entry into the nucleus or its nuclear function. In contrast, mutation of the ATPase (G227D) or the substrate-binding domain (T453D), while having no effect on GRP78 nuclear entry, these mutants exhibited diffused nuclear staining and impaired transcriptional activity, implying that the integrity of these domain functions is critical for GRP78 nuclear function.

Notably, we also discovered a novel interaction between GRP78 and GRP94 in the nucleus, marking the first report of GRP94's nuclear localization in stressed cells. This interaction, observed in both human embryonic kidney and lung cancer cells, suggests that GRP78 and GRP94 form a chaperone complex in the nucleus, mirroring their established cooperation in the ER. The interaction is dependent on the ATP-binding and substrate-binding functions of GRP78, as mutants deficient in these activities fail to colocalize with GRP94 in the nucleus. Interestingly, HSP70 and HSP90 which are the cytosolic homologs of GRP78 and GRP94, respectively, translocate to the nucleus under heat shock forming multiprotein complexes (41, 42). Our novel observation that GRP78 and GRP94, both with primary residence in the ER, can colocalize in the nucleus of the stressed and cancer cells suggests that ER stress activates a yet to be defined process in the ER that enables GRP78 efflux to the cytosol and entry into the nucleus, where it forms complex with GRP94 which also traffics to the nucleus upon ER stress. Interestingly, a mutation in the cochaperone binding site of GRP78 does not disrupt GRP78-GRP94 nuclear interaction. This raises the question whether their nuclear association follows the same or different interactions as in the ER, which remains to be determined. It is possible that other ER chaperones, yet to be discovered, may also contribute to the formation of the discrete nuclear foci. In binding and sequestering ID2 and likely other transcriptional factors, nuclear GRP78 rewires the cells' transcriptome, impacting cellular homeostasis and tumorigeneses, and other diseases where GRP78 is upregulated. Our findings offer new insights into the diverse regulation and functions of GRP78 and support vigorous future investigations into the mechanisms and consequences of ER chaperone translocation to the nucleus and targeting GRP78 nuclear activities for therapeutic applications.

## Experimental procedures

### Cell lines and culture conditions

The major cell lines used in this study were as follows. Human non–small cell lung cancer cell line H1838 was kindly provided by Prof. Steven M. Dubinett (University of California, Los Angeles). Human embryonic kidney cells HEK293AD was provided by Prof. Xiang-Lei Yang (Scripps Research). The generation of the SEL1L^−/−^, and HRD1^−/−^ HEK293T cells cell lines has been previously described (30, 38). Briefly, HEK293T cells, obtained from American Type Culture Collection, were cultured at 37 °C with 5% CO_2_ in Dulbecco's modified Eagle medium with 10% fetal bovine serum (FBS) (Thermo Fisher Scientific). sgRNA oligonucleotides designed for human SEL1L (5′-GGCTGAACAGGGCTATGAAG-3′) or human HRD1 (5′-GGACAAAGGCCTGGATGTAC-3′) were inserted into lentiCRISPR, version 2 (Addgene 52961). Cells grown in 10 cm dishes were transfected with indicated plasmids using 5 μl of 1 mg/ml polyethylenimine (MilliporeSigma) per 1 μg of plasmids for HEK293T cells. The cells were cultured 24 h after transfection in medium containing 2 μg/ml puromycin for 24 h and then in normal growth medium. H1838 cells were cultivated in RPMI-1640 Medium (Corning Inc) supplemented with 10% fetal bovine serum (FBS; GeminiBio) and 1% penicillin/streptomycin (pen/strep; Corning Inc). HEK293AD, HEK293T WT, SEL1L^−/−^, and HRD1^−/−^ cells were cultivated in Dulbecco's modified Eagle medium (Corning Inc) supplemented with 10% FBS and 1% pen/strep. Cells were grown at 37 °C in a humidified atmosphere of 5% CO_2_ and 95% air. The cell lines were routinely tested for *mycoplasma* contamination.

### Compounds and treatment conditions

The ER-stress inducer Tg was purchased from Cayman Chemical. The compound was dissolved in DMSO. The final concentration of DMSO in cell culture was either 0.1% or 1%. To induce ER stress, the cells were treated with Tg at 300 nM. For all experiments, DMSO was used as vehicle control.

### Antibodies for immunoblots

The following antibodies were used in this study. Primary antibodies: mouse anti-GRP78 antibody (1:1000, BD Biosciences, 610979), rat anti-GRP94 antibody (1:1000, Enzo Life Sciences, SPA-851), rabbit anti-calnexin antibody (1:2000, Enzo Life Sciences, ADI-SPA-860), mouse anti-FLAG M2 antibody (1:2000, MilliporeSigma, F1804), mouse anti-FLAG M1 antibody (1:2000, MilliporeSigma, F3040), mouse anti-GAPDH antibody (1:5000, Santa Cruz Biotechnology, Inc, sc-32233), rabbit anti-Histone H3 antibody (1:1000, Santa Cruz Biotechnology, sc-10809), rabbit anti-SEL1L (1:10,000, home-made, (43), rabbit anti-HRD1 (1:2000, Proteintech, 13473–1), rabbit anti-OS9 (1:5000, Abcam, ab109510), rabbit anti-CD147 (1:3000, Proteintech, 11,989–1), mouse anti-HSP90 (1:5000, Santa Cruz Biotechnology Inc, sc-13119). Secondary antibodies: horseradish peroxidase (HRP) conjugated goat anti-mouse (sc-2005), goat anti-rabbit (sc-2004), goat anti-rat (sc-2006) antibodies (1:1000, Santa Cruz Biotechnology, Inc), mouse IgGκ binding protein conjugated to HRP (1:1000, Santa Cruz Biotechnology, Inc, sc-516102), and mouse anti-rabbit IgG conjugated to HRP (1:1000, Santa Cruz Biotechnology, Inc, sc-2357). Protein bands were visualized by ChemiDoc XRS+ imager (Bio-Rad Laboratories) and quantified by Image Lab (https://www.bio-rad.com/en-us/product/image-lab-software?ID=KRE6P5E8Z) Software Version 4.0.1 build 6 (Bio-Rad Laboratories).

### Plasmids, recombinant proteins, and transfection

The construction of the FLAG-GRP78 WT and mutants (G227D, R197H, T453D, and NLS^Mut^) expression vectors has been described previously (10, 17, 44). The FLAG-GRP78 expression vector lacking the ER signal sequence (F-78-ΔERS) was purchased from VectorBuilder. The ID2-Myc-FLAG expression construct was described previously (17). The FLAG-NCKX3 expression vector was purchased from Addgene (Plasmid #75206). The pcDNA3 empty vector was used as control for all experiments with overexpression. Transfection of plasmids was performed with Lipofectamine 3000 (Thermo Fisher Scientific, Cat# L3000015) following the manufacturer's instructions. The recombinant GRP78 proteins with or without the ER signal peptide were custom ordered and purchased from GenScript Biotech.

### RT-qPCR

For RNA extraction, cells were washed three times with PBS and lysed directly with 1 ml of TRI Reagent (MilliporeSigma) following the manufacturer's instructions. Two micrograms of purified RNA were reverse transcribed using qScript XLT cDNA SuperMix (Quantabio) following the manufacturer's instructions. One microliter of the resulting complementary DNA was used in real-time PCR (35 cycles: 30 s at 95 °C, 30 s at 58 °C, 45 s at 72 °C). Samples were tested in triplicate using the SYBR Green Super mix (Bio-Rad Laboratories) on the Stratagene MX3000P Real-Time QPCR System (Agilent). The primers used for each gene are as follows:

*EGFR*:

Forward: 5′-AACACCCTGGTCTGGAAGTACG-3′

Reverse: 5′-TCGTTGGACAGCCTTCAAGACC-3′

*GRP78*:

Forward: 5′-GGTGAAAGACCCCTGACAAA-3′

Reverse: 5′-GTCAGGCGATTCTGGTCATT-3′

*COL1A2*:

Forward: 5′-CTGCTGGAAGTCGTGGTGAT-3′

Reverse: 5′-ACGAAGCCCTTCTTTCCCAG-3′

*LRP1*:

Forward: 5′-CTGGCGAACAAACACACTGG-3′

Reverse: 5′-CACGGTCCGGTTGTAGTTGA-3′

*HSP90B1*:

Forward: 5′-GCCAGTTTGGTGTCGGTTTC-3′

Reverse: 5′-GGGTAATTGTCGTTCCCCGT-3′

*β-actin*:

Forward: 5′-TACCACAGGCATTGTGATGG-3′,

Reverse: 5′-TTTGATGTCACGCACGATTT-3′.

### Immunoblot analysis

Cells were lysed with ice cold radioimmunoprecipitation assay (RIPA) buffer (50 mM Tris–HCl, 150 mM NaCl, 1% NP-40, 0.5% sodium deoxycholate, and 0.1% SDS) supplemented with a Protease and Phosphatase Inhibitor Cocktail (Thermo Fisher Scientific). The lysates were incubated on ice for 30 min, and then centrifuged at 13,000 RPM for 15 min to remove debris. The resulting supernatant containing soluble proteins was collected for subsequent immunoblot analysis. Proteins were separated on 8%, 10%, or 15% SDS-PAGE gels and transferred onto nitrocellulose membranes (Bio-Rad Laboratories). The membranes were blocked with 5% nonfat dried milk dissolved in Tris-buffered saline with 0.05% Tween-20 (TBST) followed by incubation with primary antibody diluted in 5% bovine serum albumin TBST at room temperature for 2 h or overnight at 4 ^°^C. For the FLAG M1, the primary antibody was diluted in TBST buffer containing 1 mM CaCl_2_. The membranes were washed with TBST three times for 5 min each followed by incubation with HRP-conjugated secondary antibody diluted in TBST for 2 h at room temperature. Then the membranes were washed with TBST three times for 5 min each and developed with SuperSignal West Pico PLUS Chemiluminescent Substrate (Thermo Fisher Scientific). Protein bands were visualized using the ChemiDoc XRS+ imaging system (Bio-Rad Laboratories) and quantified with Image Lab Software Version 4.0.1 build 6 (Bio-Rad Laboratories).

For detection of protein levels of SEL1L, HRD1, OS9, CD147, and HSP90, cells were harvested and snap-frozen in liquid nitrogen. The proteins were extracted by sonication in NP-40 lysis buffer (50 mM Tris–HCl at pH 7.5, 150 mM NaCl, 1% NP-40, and 1 mM EDTA) with protease inhibitor (MilliporeSigma), DTT (MilliporeSigma, 1 mM), and phosphatase inhibitor cocktail (MilliporeSigma). Lysates were incubated on ice for 30 min and centrifuged at 16,000*g* for 10 min. Supernatants were collected and analyzed for protein concentration using Bio-Rad Protein Assay Dye (Bio-Rad Laboratories). Subsequently, 10 to 30 μg of protein was denatured at 95 °C for 5 min in 5 × SDS sample buffer (250 mM Tris–HCl pH 6.8, 10% sodium dodecyl sulfate, 0.05% bromophenol blue, 50% glycerol, and 1.44 M β-mercaptoethanol). Protein was separated using SDS-PAGE followed by electrophoretic transfer to polyvinylidene fluoride (Thermo Fisher Scientific) membrane. The blots were incubated in 2% bovine serum albumin/TBST with the primary antibodies overnight at 4 °C. Membranes were washed with TBST and incubated with HRP-conjugated secondary antibodies (Bio-Rad Laboratories, 1:10,000) at room temperature for 1 h for enhanced chemiluminescence Detection System (Bio-Rad Laboratories) development.

### Immunofluorescence

Cells were seeded on Millicell EZ SLIDE (MilliporeSigma, PEZGS0816) and allowed to attach overnight. Next day, the cells were transfected or treated with Tg as indicated for 24 h. Then the media were removed, and the cells were washed with PBS three times and fixed with 4% paraformaldehyde and permeabilized in 0.02% Triton X-100 for 10 min at room temperature. After incubation with blocking buffer (5% bovine serum albumin, 0.1% Tween-20, and PBS) for 1 h, cells were incubated at 4 °C overnight with primary antibodies diluted in Phosphate-Buffered Saline with Tween-20 (PBST) in a humidified chamber at 4 °C. Primary antibody: mouse anti-GRP78 (1:500, MAb159, Gift from Dr Parkash Gill, USC); mouse anti-FLAG M2 antibody (1:2000, MilliporeSigma, F1804); and rat anti-GRP94 antibody (1:1000, Enzo Life Sciences SPA-851). Cells were washed three times with PBS and were incubated with Alexa Fluor-conjugated secondary antibodies for 1 h at room temperature, followed by three more washes with PBS. Secondary antibody: Alexa Fluor 488 goat anti-mouse antibody (1:500, Thermo Fisher Scientific, #A-11001) and Alexa Fluor 594 donkey anti-rat antibody (1:500, Thermo Fisher Scientific, #A-21209). Cells were mounted with VECTASHIELD Antifade Mounting Medium with DAPI (Vector Laboratories, Inc, #H1200). Cell images were acquired with Leica SP8 LIGHTNING Confocal Microscope using a 63 × oil objective. All immunofluorescence experiments were repeated 2 times and at least 10 cells were examined. To measure the colocalization of GRP78 and GRP94 in the nucleus, the GRP78-GRP94 costaining images were analyzed by Coloc 2 plugin (Mander's O.C.) in the FIJI-ImageJ software (https://imagej.net/ij/) (n = 10).

### Subcellular fractionation

HEK293AD cells were seeded at a density of 5 × 10^6^ cells in a 10 cm dish and treated as described 24 h before collection in 500 μl cell fractionation buffer (20 mM Hepes, pH 7.5, 10 mM KCl, 2 mM MgCl_2_, 2 mM EDTA, 1 mM DTT, and protease inhibitor). Briefly, 100 μl was taken as whole cell lysate. The remaining cells were passed 10 times through a 27-gauge needle. Nuclei and cytoplasm were separated by centrifugation at 750*g* for 5 min. The supernatant was taken as cytoplasmic fraction, and the nuclear pellet washed three times with cell fractionation buffer. Cold lysis buffer (0.5% NP-40, 50 mM Tris–HCl, pH 7.5, 100 mM NaCl, 0.1 mM EDTA, 10% glycerol, and 1 mM DTT) was added to all fractions. Complete lysis was achieved by freezing and thawing. Lysates were spun down at 14,000*g* for 20 min to remove insoluble components, and SDS loading buffer was added before Western blot analysis.

### Co-immunoprecipitation

HEK293AD cells were singly transfected with F-78 (WT), or cotransfected with ID2-FLAG-Myc and F-78 constructs. After 24 h of transfection, cells were lysed by sonication in immunoprecipitation buffer (20 mM Tris–HCl pH 7.5, 150 mM NaCl, 2 mM EDTA, and 0.1% Triton X-100) supplemented with protease inhibitor cocktail (Roche). Cell lysates were centrifuged at 13,000 RPM for 30 min at 4 °C, and the clarified supernatant was saved. Immunoprecipitation was carried out by incubating the supernatants with anti-FLAG (M2) or anti-Myc antibody (Thermo Fisher Scientific, 9E10) for 1 h at room temperature, followed by incubation with protein A agarose magnetic beads (Thermo Fisher Scientific) at 4 °C overnight. The beads were washed three times with immunoprecipitation buffer, and the immunoprecipitated proteins were eluted in 2X SDS sample buffer and detected by Western blot.

### Statistical analysis

All graphs were produced with GraphPad Prism version 10.0 (GraphPad Software). Data were presented as means ± SD. All comparisons were analyzed by unpaired 2-tailed Student's *t* test using Microsoft Excel with the Bonferroni correction (*p* adjust = *p* original X number of treatment groups) applied to control the family wise error rate. A *p*-adjust value of ≤ 0.05 is signified by ∗, ≤ 0.01 by ∗∗, ≤ 0.001 by ∗∗∗, and *p*-adjust value of ≤ 0.0001 by ∗∗∗∗, n.s. denotes not significant.

## Data availability

All data generated and analyzed during this study are included in this published article.

## Supporting information

This article contains supporting information.

## Conflict of interest

A. S. L. is a scientific advisory board member of BiPER Therapeutics. The other coauthors declare that they have no conflicts of interest with the contents of this article.

## Supporting information

Supplmental Information
