# Supplementary material for: Requirements for nuclear GRP78 transcriptional regulatory activities and interaction with nuclear GRP94
Source: J Biol Chem. 2025 Feb 28;301(4):108369. doi: 10.1016/j.jbc.2025.108369 (PMC11997380; doi:10.1016/j.jbc.2025.108369)
Supplement: Supplmental Information [file mmc1.docx]

**Supplemental Figure S1**


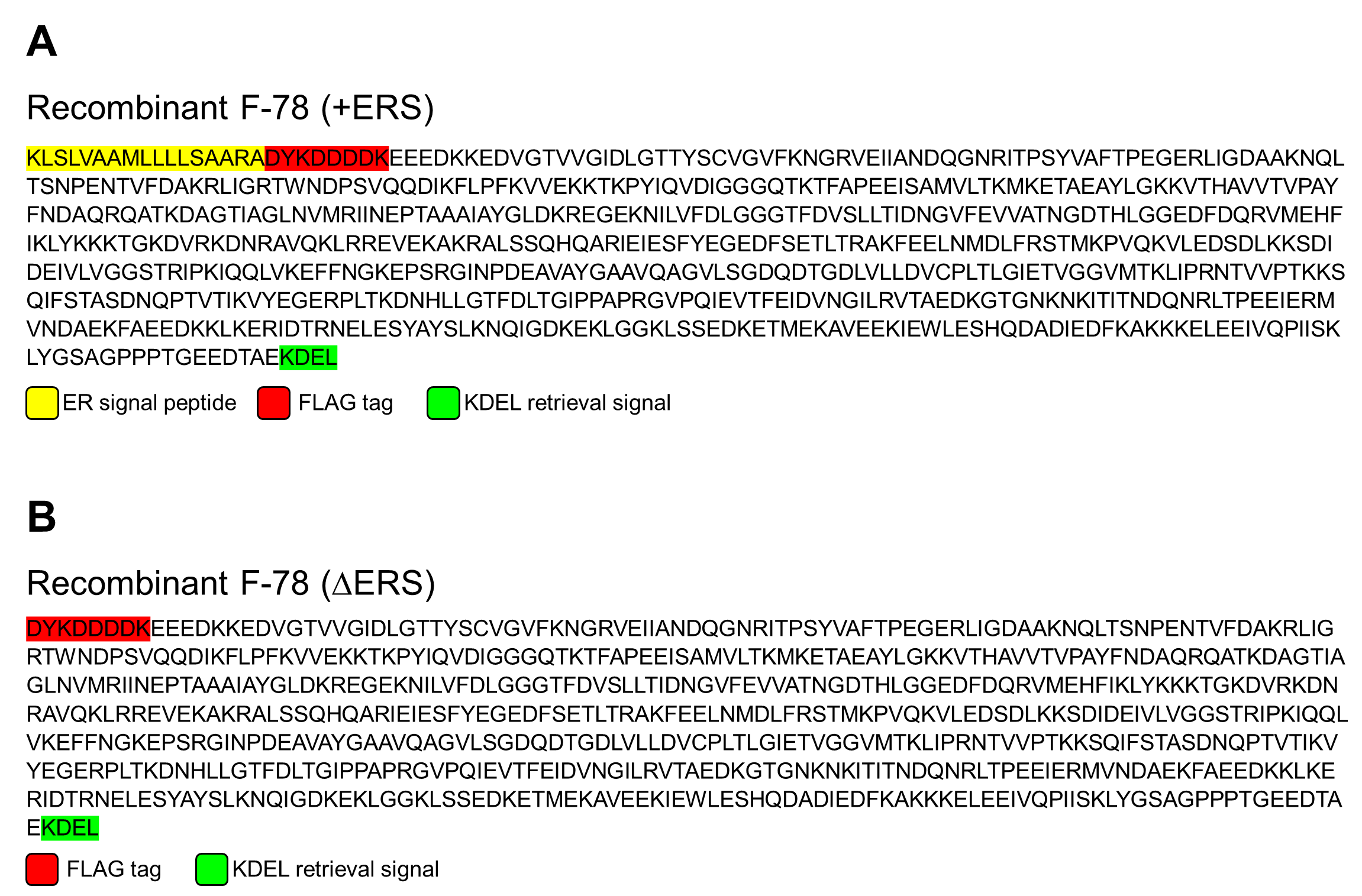


**Figure S1. Amino acid sequence of recombinant F-78 proteins.** Amino acid sequence of F-78 protein with the ER signal peptide (+ERS) (A) or without (ΔERS) (B). Yellow color: ER signal peptide, red color: FLAG tag, and green color: KDEL retrieval signal.

**Supplemental Figure S2**


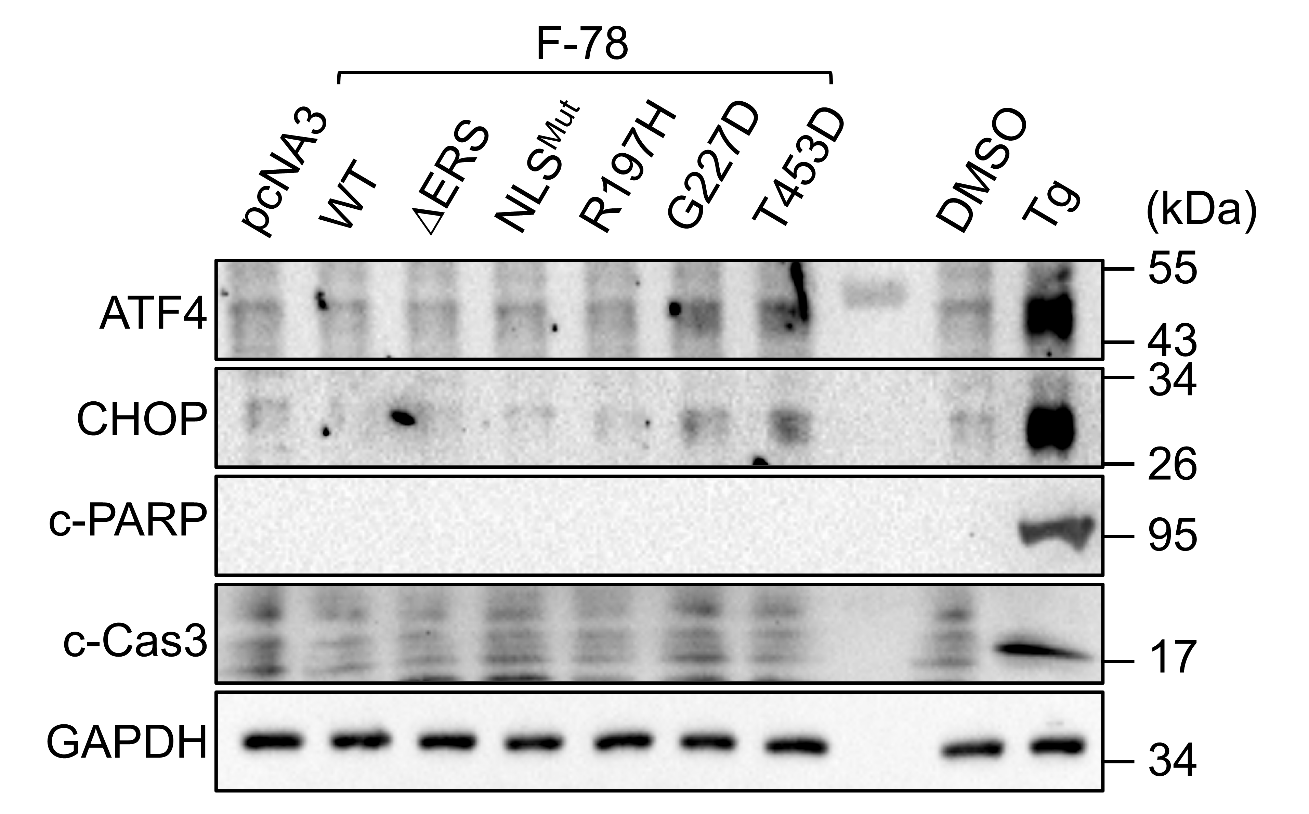


**Figure S2. Effects of overexpression of wild type and mutant forms of GRP78 on the UPR and the onset of apoptosis**. HEK293AD cells were transfected with the empty vector pcDNA, expression vectors for F-78 (WT) or F-78 mutants(∆ERS, NLS^Mut^, R197H, G227D, or T453D) for 48 h and either treated with DMSO or Tg for 24 h. Whole cell lysates were subjected to Western blot analysis for ATF4, CHOP, cleaved PARP (c-PARP), or cleaved Caspase-3 (c-Cas3) with GAPDH serving as loading control.
